# Suppression of Breast Cancer Cell Migration by Small Interfering RNA Delivered by Polyethylenimine-Functionalized Graphene Oxide

**DOI:** 10.1186/s11671-016-1463-0

**Published:** 2016-05-12

**Authors:** Yuan-Pin Huang, Chao-Ming Hung, Yi-Chiang Hsu, Cai-Yan Zhong, Wan-Rou Wang, Chi-Chang Chang, Mon-Juan Lee

**Affiliations:** Department of Cosmetics and Fashion Styling, Cheng Shiu University, Kaohsiung, Taiwan; Department of General Surgery, E-Da Hospital, Kaohsiung, Taiwan; Graduate Institute of Medical Sciences, Chang Jung Christian University, Tainan, Taiwan; Innovative Research Center of Medicine, Chang Jung Christian University, Tainan, Taiwan; Department of Bioscience Technology, Chang Jung Christian University, No. 1 Changda Rd., Gueiren District, Tainan City, 71101 Taiwan; Department of Obstetrics and Gynecology, E-Da Hospital, Kaohsiung, Taiwan

**Keywords:** Graphene oxide (GO), Polyethylenimine (PEI), Small interfering RNA (siRNA), C-X-C chemokine receptor type 4 (CXCR4), Cancer cell migration

## Abstract

The carbon-based nanomaterial graphene can be chemically modified to associate with various molecules such as chemicals and biomolecules and developed as novel carriers for drug and gene delivery. In this study, a nonviral gene transfection reagent was produced by functionalizing graphene oxide (GO) with a polycationic polymer, polyethylenimine (PEI), to increase the biocompatibility of GO and to transfect small interfering RNA (siRNA) against C-X-C chemokine receptor type 4 (CXCR4), a biomarker associated with cancer metastasis, into invasive breast cancer cells. PEI-functionalized GO (PEI-GO) was a homogeneous aqueous solution that remained in suspension during storage at 4 °C for at least 6 months. The particle size of PEI-GO was 172 ± 4.58 and 188 ± 5.00 nm at 4 and 25 °C, respectively, and increased slightly to 262 ± 17.6 nm at 37 °C, but remained unaltered with time. Binding affinity of PEI-GO toward siRNA was assessed by electrophoretic mobility shift assay (EMSA), in which PEI-GO and siRNA were completely associated at a PEI-GO:siRNA weight ratio of 2:1 and above. The invasive breast cancer cell line, MDA-MB-231, was transfected with PEI-GO in complex with siRNAs against CXCR4 (siCXCR4). Suppression of the mRNA and protein expression of CXCR4 by the PEI-GO/siCXCR4 complex was confirmed by real-time PCR and western blot analysis. In addition, the metastatic potential of MDA-MB-231 cells was attenuated by the PEI-GO/siCXCR4 complex as demonstrated in wound healing assay. Our results suggest that PEI-GO is effective in the delivery of siRNA and may contribute to targeted gene therapy to suppress cancer metastasis.

## Background

Graphene oxide (GO) is a carbon-based nanomaterial with a single layer of carbon molecules covalently linked to oxidized functional groups such as carboxyl (–COOH) and hydroxyl (–OH) groups, which can be chemically modified to increase its biocompatibility in order to associate with drugs and biomolecules [1]. Functionalized graphene oxide has been considered nanocarriers for drug delivery [2–4], gene delivery [5–8], combined delivery of drug and gene for cancer therapy [9–12], as well as protein transportation [13–15]. It has also been applied in biosensing [16, 17], bioimaging [18, 19], and tissue engineering [20, 21].

Polyethylenimine (PEI), a polycationic polymer that attracts nucleic acids through electrostatic interaction, is commonly used in the functionalization of nanomaterials for gene delivery. PEI-functionalized GO (PEI-GO) successfully delivered both small interfering RNA (siRNA) and anticancer drugs to enhance chemotherapeutic effect in cancer cells [9]. A noncovalent PEI-GO complex was reported to enhance GFP plasmid expression in HeLa cells [22]. By exploiting the near-infrared (NIR) optical absorbance of GO, photothermally controlled delivery of siRNA and plasmid DNA by GO functionalized with both PEI and polyethylene glycol (PEG) was achieved [23, 24].

Chemokine (C-X-C motif) receptor 4 (CXCR4), which is a class-A G protein-coupled receptor (GPCR), has been considered a biomarker for cancer metastasis and poor prognosis [25, 26]. Suppression of CXCR4 and its signaling axis is therefore a common strategy to inhibit cancer cell migration and metastasis [27–30]. Drug and gene delivery systems based on nanomaterials are thus designed to target against CXCR4 and facilitate cancer therapy and imaging. Anti-CXCR4 monoclonal antibody conjugated to superparamagnetic nanoparticles was applied in molecular imaging of pancreatic cancer cell lines [31]. PEG-functionalized carbon nanotube and cationic dextran-based nanoparticles were reported to deliver siRNA against CXCR4 into primary cells and animal model for colorectal cancer [32, 33]. Peptide ligands and peptide dendrimers against CXCR4 were used alone or in conjugation with nanoparticles to deliver anticancer drugs, inhibit tumor metastasis, and enhance molecular imaging [34–37]. In addition, synthetic polycationic viologen dendrimers (VGD) targeting CXCR4 were also developed to facilitate targeted delivery of plasmid DNAs and cancer therapy [38].

In this study, we explored the potential of PEI-GO in the transfection of siRNAs against CXCR4 (siCXCR4) to suppress the migration of MDA-MB-231 cells, a metastatic cancer cell line overexpressing CXCR4. Transfection efficiency was evaluated by the level of suppression of CXCR4 mRNA, as well as the migration ability of MDA-MB-231, and was compared to a commercial transfection reagent, Lipofectamine 2000. Our results suggest that PEI-GO is a potentially efficient nonviral transfection reagent that may contribute to targeted cancer therapy.

## Methods

### PEI Functionalization of Graphene Oxide

Graphene oxide (GO, Sigma-Aldrich, St. Louis, MO, USA) was activated with (1-ethyl-3-(3-dimethyl-aminopropyl) carbodiimide (EDC) and linked to PEI (branched, average *M*_W_~25,000 by LS, average *M*_n_~10,000 by GPC, Sigma-Aldrich, St. Louis, MO, USA) through the formation of amide bonds (–CONH–) using methods reported in the literature [9]. To remove unbound PEI, the reaction product was washed with ddH_2_O and centrifuged repeatedly at 3,000 rpm for 15–30 min in an Amicon Ultra-15 Centrifugal Filter Unit (Millipore, Billerica, MA, USA) with a molecular weight cut-off of 100 kDa. The flow-through was subjected to ninhydrin assay to determine the level of free PEI.

### Ninhydrin Assay

During washing of PEI-GO, 1 ml of flow-through from the Amicon Ultra-15 Centrifugal Filter Unit was mixed with 200 μl of 2 % (*w*/*v*) ninhydrin solution, followed by reaction in a boiling water bath for 3 min. Ninhydrin reacts with the primary and secondary amines of free PEI to produce Ruhemann’s purple, which was gradually decreased with successive washing. PEI-GO was considered free of unreacted PEI when the color of Ruhemann’s purple was undetectable by the naked eye.

### Characterization of PEI-GO

The difference in morphology between pristine GO and PEI-GO was examined by transmission electron microscopy (JEOL 2000FX TEM) and scanning electron microscopy (JSM-6500F SEM). The particle size and zeta potential of PEI-GO were determined by dynamic light scattering using Zetasizer Nano ZS system (Malvern Instruments, Worcestershire, UK).

### Electrophoretic Mobility Shift Assay (EMSA)

Dharmacon siGENOME GAPD Control siRNA (Thermo Fisher Scientific, Waltham, MA, USA) was used in EMSA to analyze the binding capacity of PEI-GO. The PEI-GO:siRNA complex was formed by incubating 0–0.6 μg of PEI-GO with 0.2 μg siRNA at various mass ratios in serum-free cell culture medium for 20 min at room temperature. The complex was then mixed with SYBR Green I and resolved by 1 % agarose gel as described previously [39].

### Cell Culture

Human breast carcinoma cell line MDA-MB-231 was cultured at 37 °C in the absence of CO_2_ in Leibovitz’s L-15 medium (Gibco, Life Technologies, Carlsbad, CA, USA) supplemented with 10 % fetal bovine serum (FBS), 50 units/ml penicillin, and 50 μg/ml streptomycin. The medium was refreshed every 3–4 days, and confluent cells were subcultured 7 days after seeding. Cells were seeded at 5000 cells/well in 96-well plates for cell viability assay, at 10^5^ cells/well in 6-well plates for total RNA extraction, and at 2 × 10^4^ cells/well in 24-well plates for wound healing assay.

### Cell Viability Assay

MDA-MB-231 cells were treated with 0–20 μg/ml PEI-GO for 48 h, followed by WST-1 assay using Quick Cell Proliferation Colorimetric Assay Kit (BioVision, Milpitas, CA, USA). Cell viability was quantitated spectrophotometrically by measuring the optical density at 450 nm, with a reference wavelength of 650 nm.

### siRNA Transfection with PEI-GO

MDA-MB-231 cells were treated with Dharmacon siGENOME siRNA specific for human CXCR4 (siCXCR4) or siGENOME GAPD Control siRNA (siMOCK). The siRNA was delivered either by Lipofectamine 2000 (Life Technologies, Carlsbad, CA, USA) according to manufacturer’s instructions or by PEI-GO. PEI-GO was incubated with siCXCR4 at mass ratios of 0.3:1, 0.5:1, and 1:1 for 20 min at room temperature before cultured with MDA-MB-231 cells to achieve a final siCXCR4 concentration of 25 nM. Two days after siRNA transfection, cells were harvested for RNA extraction and real-time PCR analysis or subjected to wound healing assay.

### Real-Time Polymerase Chain Reaction

Total RNA was isolated by PureLink® RNA Mini Kit (Thermo Fisher Scientific, Waltham, MA, USA), and cDNA synthesis was carried out using the SuperScript III First-Strand Synthesis SuperMix for qRT-PCR (Life Technologies, Carlsbad, CA, USA) according to the manufacturer’s instructions. The cDNA was diluted to a final concentration of ~1 ng/μl and reacted with CXCR4 (NM_003467)- or GAPDH (NM_002046.4)-specific primer pairs and QuantiFast SYBR® Green PCR Kit (QIAGEN, Germantown, MD, USA). PCR was performed by Applied Biosystems 7300 Real-Time PCR System and monitored with Applied Biosystems Sequence Detection Software V1.2 (Life Technologies, Carlsbad, CA, USA) as described previously [40].

### Wound Healing Assay

Wound healing assay was performed by following the protocol provided in the literature [41]. MDA-MB-231 cells were cultured in a 24-well plate and treated with siCXCR4 or siMOCK complexed with either PEI-GO or Lipofectamine 2000 for 48 h. The cell monolayer in each well was scraped with a p200 pipet tip to create a gap. After washing with culture medium to remove cell debris, the cells were allowed to migrate for 24 h, followed by observation under an Olympus CKX41 optical microscope.

### Statistical Analysis

Statistical analysis was performed on data from at least three independent experiments. Significant difference relative to the control was tested using Student’s *t* test. Levels of significance of *p* < 0.05 and 0.01 were accepted as significant and highly significant, respectively.

## Results

### Characterization of PEI-GO

**Fig. 1:**
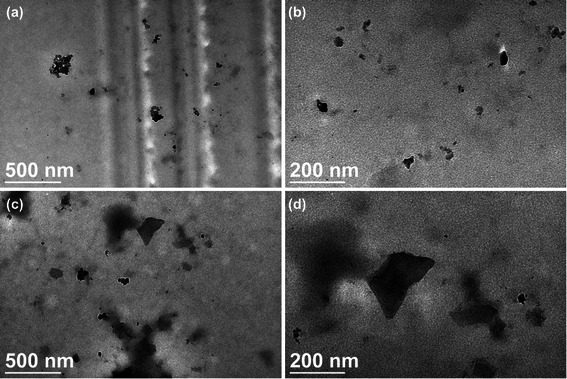
Transmission electron microscopy images of pristine GO and PEI-GO. The surface morphology of pristine GO (**a**, **b**) was compared with that of PEI-GO (**c**, **d**) by a JEOL 2000FX TEM at different scales

**Fig. 2:**
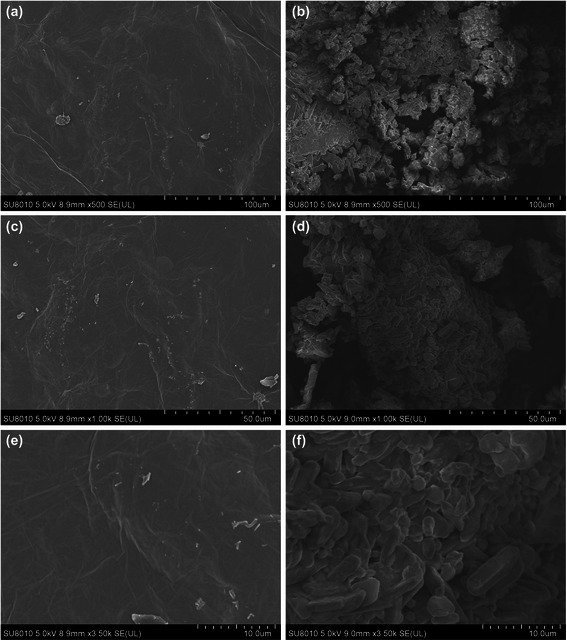
Scanning electron microscopy images of pristine GO and PEI-GO. The surface morphology of pristine GO (**a**, **c**, **e**) was compared with that of PEI-GO (**b**, **d**, **f**) by a JSM-6500 F SEM at different scales

**Fig. 3:**
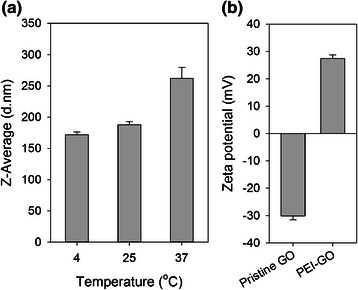
Average particle size and zeta potential of PEI-GO analyzed by dynamic light scattering. **a** The average particle diameter of 1 mg/ml PEI-GO was compared at 4, 25, and 37 °C. **b** The zeta potential of 1 mg/ml PEI-GO was compared to that off pristine GO at 25 °C and neutral pH. *Error bars* represent standard deviations (*n* ≥ 3)

PEI functionalization increased the hydrophilicity and dispersibility of GO, which formed aggregates and precipitated in water prior to functionalization. The PEI-GO suspension can be maintained for at least 10 months without precipitation. As examined by transmission electron microscopy and scanning electron microscopy, pristine GO was tightly packed (Fig. 1a, b) and had a relatively smooth surface (Fig. 2a, c, e). PEI functionalization increased the surface area of PEI-GO, as well as the spacing between graphene layers, which appeared more extended (Fig. 1c, d) and was highly agglomerated, indicating that the stacking of the graphene sheets was disturbed (Fig. 2b, d, f). The particle size of PEI-GO was 172 ± 4.58 and 188 ± 5.00 nm at 4 and 25 °C, respectively, and increased slightly to 262 ± 17.6 nm at 37 °C (Fig. 3a), suggesting that that PEI-GO may be partially aggregated in cell culture. However, when the particle size immediately after synthesis was compared to that after stored at 4 °C for over 10 months, no significant change was observed (data not shown). As shown in Fig. 3b, the zeta potential of pristine GO was negative (−30.2 ± 1.34 mV), while that of PEI-GO was positive (27.4 ± 1.25 mV), indicating that PEI functionalization increased the positive charge on the surface of GO and contributed to the electrostatic repulsion that stabilized the PEI-GO suspension.

### Binding Capacity of PEI-GO to siRNA

**Fig. 4:**
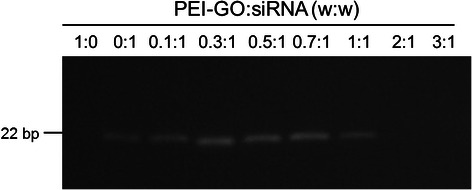
Binding capacity of PEI-GO toward siRNA. PEI-GO was reacted with Dharmacon siGENOME GAPD control siRNA at various mass ratios, followed by electrophoretic mobility shift assay (EMSA)

Binding capacity of PEI-GO toward siRNA was assessed by electrophoretic mobility shift assay (EMSA). PEI-GO was complexed with siRNA at various mass ratios and resolved with agarose gel electrophoresis (Fig. 4). Binding of siRNA to PEI-GO resulted in reduced mobility of free siRNAs and their availability for SYBR Green I intercalation. As the amount of PEI-GO increased, more siRNAs were adsorbed, resulting in decreased fluorescence signal of free siRNAs. The migration of siRNA was completely inhibited when the mass ratio of PEI-GO:siRNA was 2:1 and above.

### Cytotoxicity of PEI-GO

**Fig. 5:**
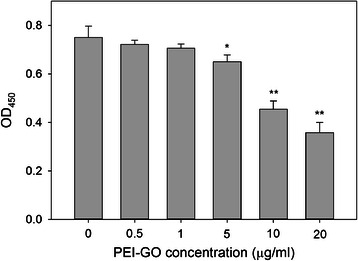
Cytotoxicity of PEI-GO in MDA-MB-231 cells. Human breast carcinoma cells MDA-MB-231 were treated with 0–20 μg/ml of PEI-GO for 48 h. Cell viability was determined by WST-1 assay and quantitated spectrophotometrically by measuring the optical density at 450 nm, with a reference wavelength of 650 nm. *Error bars* represent standard deviations (*n* ≥ 3). *p < 0.05 and **p < 0.01 compared to the control

The cytotoxicity of PEI-GO in MDA-MB-231 cells, a invasive breast cancer cell line, was analyzed by WST-1 assay. After incubated with PEI-GO for 48 h, we observed that the viability of MDA-MB-231 cells decreased with increasing concentrations of PEI-GO (Fig. 5). In the presence of 20 μg/ml PEI-GO, the number of viable cells reduced to 47.6 % of that of the control. The final concentration of PEI-GO in siRNA transfection was therefore limited within the range which had no significant effect on cell viability.

### Suppression of CXCR4 by siCXCR4 Transfected with PEI-GO

**Fig. 6:**
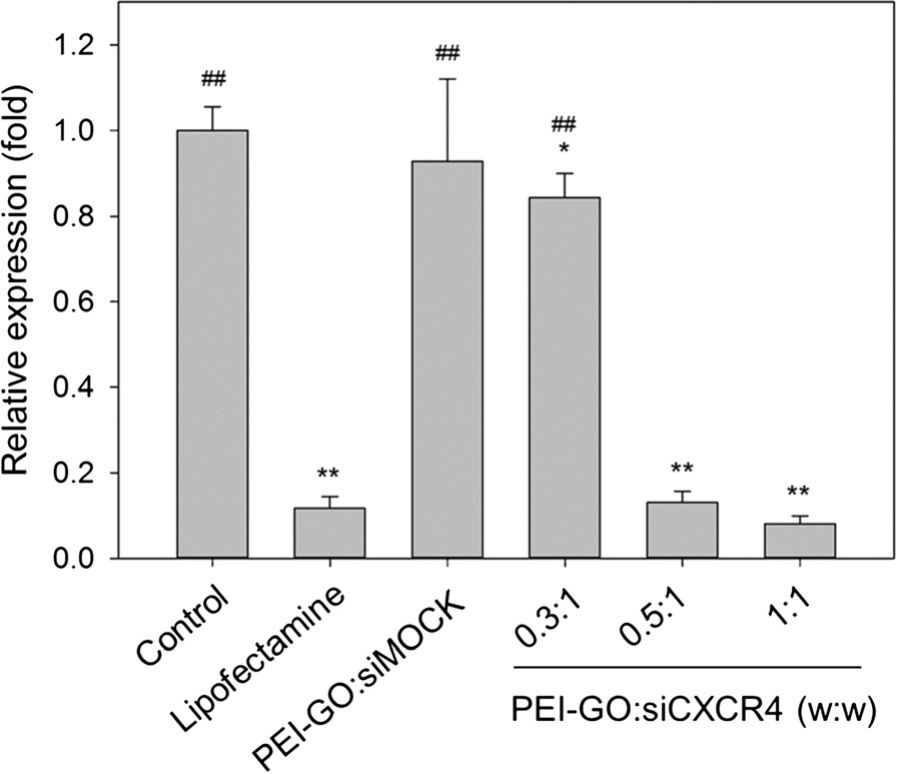
Relative CXCR4 mRNA expression of MDA-MB-231 cells transfected with PEI-GO:siCXCR4 complexes. PEI-GO was incubated with siCXCR4 at mass ratios of 0.3:1, 0.5:1, and 1:1 for 20 min at room temperature before cultured with MDA-MB-231 cells to achieve a final siCXCR4 concentration of 25 nM. Two days after siRNA transfection, cells were harvested for RNA extraction and real-time PCR analysis. Control, MDA-MB-231 cells cultured in growth medium for 48 h; Lipofectamine, MDA-MB-231 cells transfected with siCXCR4 using Lipofectamine 2000 as transfection reagent; PEI-GO:siMOCK, MDA-MB-231 cells transfected with PEI-GO:siMOCK at a mass ratio of 1:1. *Error bars* represent standard deviations (*n* ≥ 3). *p < 0.05 and **p < 0.01 compared to the control; ^##^ *p* < 0.01 compared to Lipofectamine

The transfection efficiency of PEI-GO compared to Lipofectamine 2000 was demonstrated by delivering siCXCR4 into MDA-MB-231 cells. After siCXCR4 treatment for 48 h, CXCR4 mRNA expression reduced significantly to 13 and 8 % of untreated control at PEI-GO:siCXCR4 mass ratios of 0.5:1 and 1:1, respectively, but was nearly unaffected at a PEI-GO:siCXCR4 ratio of 0.3:1, and in the presence of siMOCK, a nonspecific siRNA control (Fig. 6). Transfection efficiency of PEI-GO was comparable to that of the Lipofectamine:siCXCR4 complex, which reduced CXCR4 expression to 12 % of control. These results suggest that at appropriate mass ratios, target-specific and efficient transfection can be achieved by PEI-GO.

### Effect of siCXCR4 Transfected by PEI-GO on Cell Migration

**Fig. 7:**
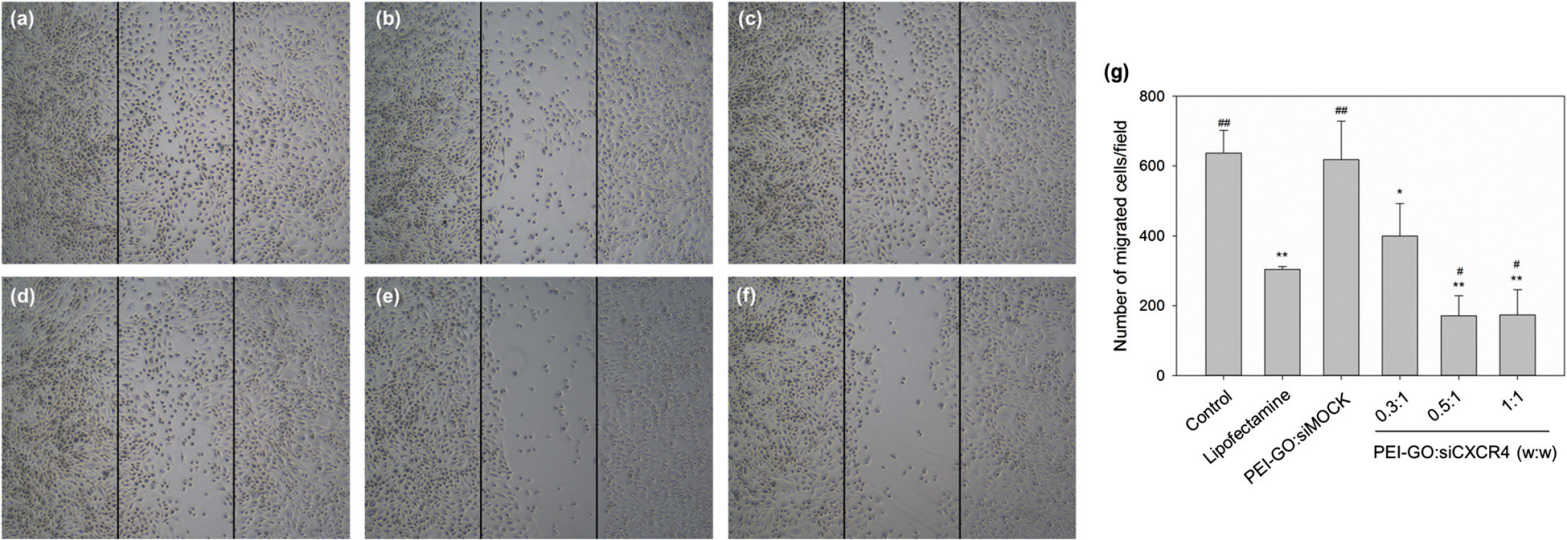
Wound healing assay of MDA-MB-231 cells transfected with PEI-GO:siCXCR4 complexes. MDA-MB-231 cells were allowed to migrate over a cell-free gap (bordered by the pair of black lines) after treated with PEI-GO:siCXCR4 complexes of mass ratios 0.3:1 (**d**), 0.5:1 (**e**), and 1:1 (**f**) for 48 h. The results were compared with untreated cells (**a**) and those treated with Lipofectamine 2000:siCXCR4 (**b**) or PEI-GO:siMOCK (**c**). Migrated cells per field from three independent experiments were quantitated as shown in the bar graph (**g**)

The effect of CXCR4 suppression on cell migration was examined by wound healing assay. MDA-MB-231 cells transfected with siCXCR4 were allowed to migrate in a cell-free gap created in the culture plate. For untreated cells or those treated with siMOCK, the gap was filled with migrated cells after 24 h (Fig. 7a, c). When the mRNA expression of CXCR4 was suppressed by siCXCR4, fewer cells were present in the gap, indicating that cell migration was retarded. As shown in Fig. 7d–f, siCXCR4 delivered by PEI-GO suppressed the migration of MDA-MB-231 cells at PEI-GO:siCXCR4 ratios of 0.5:1 and 1:1, but the effect was insignificant when the PEI-GO:siCXCR4 ratio was 0.3:1, consistent with the results of CXCR4 gene expression (Fig. 6). In addition, the extent of migrational suppression resulted from PEI-GO:siCXCR4 was comparable to that of the Lipofectamine:siCXCR4 complexes (Fig. 7g).

## Discussion

With improved vector designs, recent clinical trials have eliminated the safety concerns of gene therapy and demonstrated remarkable therapeutic benefits in inherited diseases of the blood and immune and nervous systems [42]. Gene therapy is expected to become a new approach to the development of novel therapeutic strategies beyond conventional methods. Although current strategies on clinical gene therapy are based predominantly on viral vectors, nonviral transfection reagents provide safer alternatives without potential side effects such as immunogenicity and carcinogenesis that are associated with viral transfection [43].

In this study, we demonstrated that PEI-GO is an effective nonviral carrier for siRNA delivery and may potentially be applied in targeted gene therapy to suppress cancer metastasis. Interestingly, studies have shown that pristine graphene or GO, as well as polyethylene glycol (PEG)-modified GO (PEG-GO) inhibit breast cancer cell migration through impairment of oxidative phosphorylation and mitochondrial respiration [44, 45]. In addition, GO selectively targets and retards the clonal expansion of multiple cancer stem cells [46]. These results indicate that GO alone is capable of suppressing cancer metastasis and tumor development. However, because a relatively lower concentration of PEI-GO was used in this study (0.3 μg/ml PEI-GO compared to 40 or 80 μg/ml PEG-GO in the literature), inhibition of cell migration was not observed when MDA-MB-231 cells were treated with PEI-GO alone.

## Conclusions

Our results indicate that PEI-GO is capable of delivering siCXCR4 to suppress gene expression and metastatic potential of MDA-MB-231 cells. PEI-GO may be developed as a novel nonviral transfection reagent that contributes to targeted gene therapy to suppress cancer metastasis.
